# Bilateral pleural effusion associated with remitting seronegative symmetrical synovitis with pitting edema syndrome

**DOI:** 10.1093/omcr/omac003

**Published:** 2022-02-19

**Authors:** Yoko Nagatomo, Mariko Ono, Hayato Kinoshita, Yukihisa Takeda, Hiroyuki Nakamura, Kazutetsu Aoshiba

**Affiliations:** Department of Respiratory Medicine, Tokyo Medical University Ibaraki Medical Center, Ibaraki 300-0395, Japan; Department of Respiratory Medicine, Tokyo Medical University, 6-7-1 Nishishinjuku, Shinjuku-ku, Tokyo 160-0023, Japan; Department of Respiratory Medicine, Tokyo Medical University Ibaraki Medical Center, Ibaraki 300-0395, Japan; Department of Respiratory Medicine, Tokyo Medical University, 6-7-1 Nishishinjuku, Shinjuku-ku, Tokyo 160-0023, Japan; Department of Respiratory Medicine, Tokyo Medical University Ibaraki Medical Center, Ibaraki 300-0395, Japan; Department of Respiratory Medicine, Tokyo Medical University, 6-7-1 Nishishinjuku, Shinjuku-ku, Tokyo 160-0023, Japan; Department of Respiratory Medicine, Tokyo Medical University Ibaraki Medical Center, Ibaraki 300-0395, Japan; Department of Respiratory Medicine, Tokyo Medical University Ibaraki Medical Center, Ibaraki 300-0395, Japan; Department of Respiratory Medicine, Tokyo Medical University Ibaraki Medical Center, Ibaraki 300-0395, Japan

Remitting seronegative symmetrical synovitis with pitting edema syndrome (RS3PE) is a rare elderly onset rheumatic syndrome characterized by acute-onset symmetrical distal extremity edema. Here, we report a case of RS3PE accompanying bilateral pleural effusion.

**Figure 1:**
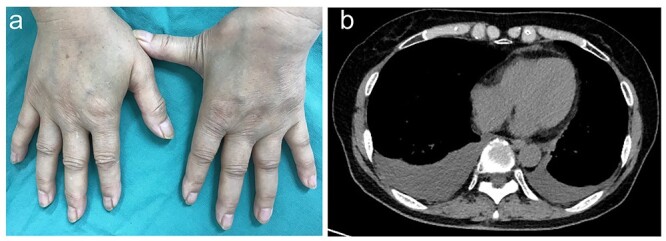
key clinical images. (**a**) Dorsal view of diffusely swollen hands with pitting edema. (**b**) Chest CT scan showing bilateral pleural effusions.

A 71-year-old woman presented to our hospital with a 2-week history of sudden bilateral chest pain onset. Physical examination revealed pitting edema in the dorsum of both hands (Fig. 1a) and tenderness of bilateral shoulder and wrist joints. Chest radiography and computed tomography (CT) scan indicated pleural effusion in both lungs (Fig. 1b). Blood test results showed elevated levels of C-reactive protein (CRP; 5.69 mg/dl; normal: < 0.3 mg/dl), vascular endothelial growth factor (VEGF: 346; normal: <38.8) and matrix metalloproteinase 3 (124; normal: <59.7). The results of rheumatoid factor test, anti-nuclear antibody and anti-cyclic citrullinated peptide antibody were negative. Remitting seronegative symmetrical synovitis with pitting edema syndrome (RS3PE) was diagnosed in accordance with the following diagnostic criteria [1–3]: (i) pitting edema in the extremities with synovitis, (ii) acute onset, (iii) age ≥ 50 years and (iv) negative findings for rheumatoid factor. One week after treatment with medium-dose prednisolone (15 mg/day), chest pain, hand edema and joint pain were completely resolved and CRP level was normalized. Chest radiography after 2 weeks treatment revealed disappearance of pleural effusions. Prednisolone dose was reduced and tapered off 21 days later. No relapse was observed in 2 months of follow-up. Findings of 18-fluoro-2-deoxyglucose positron emission tomography and CT were not suspicious for concomitant malignant tumor. However, a follow-up study should investigate occult tumors because ~20% of RS3PE cases are reportedly associated with malignancies, which may present before, during or after the diagnosis of RS3PE [3, 4]. Although pleural effusion is a rare complication of RS3PE [5, 6], a case of massive bilateral pleural effusions with pericaridial effusion requiring steroid pulse therapy has been reported [5]. Elevated VEGF levels may be associated with pleural effusion in RS3PE [5, 6]. RS3PE should be considered a possible etiology of bilateral pleural effusions.
